# Dietary Polyphenol Combinations Have a Multifaceted Inhibitory Effect on Metabolic Rewiring and Signaling Pathways in Neuroblastoma

**DOI:** 10.3390/ph18111717

**Published:** 2025-11-12

**Authors:** Natalia Karpova, Elizaveta Fefilova, Alexandra Daks, Sergey Parfenyev, Alexander Nazarov, Nick A. Barlev, Oleg Shuvalov

**Affiliations:** 1 Institute of Cytology of the Russian Academy of Sciences, St. Petersburg 194064, Russia; natalyndk@gmail.com (N.K.); e.fefilova@list.ru (E.F.); alexandra.daks@gmail.com (A.D.); gen21eration@gmail.com (S.P.); anazarov2021@gmail.com (A.N.); 2 Department of Biomedical Sciences, School of Medicine, Nazarbayev University, Astana 010000, Kazakhstan; nikolai.barlev@nu.edu.kz

**Keywords:** neuroblastoma, dietary polyphenols, metabolic reprogramming, N-Myc, c-Myc, curcumin, resveratrol, quercetin, PI3K/AKT/mTOR

## Abstract

**Background/Objectives**: Numerous studies have demonstrated that dietary plant-derived polyphenols suppress signaling and metabolic pathways in various malignancies, including neuroblastoma. In the present study, we compared the inhibitory activities of selected polyphenols and their combinations on key metabolic and signaling pathways in two human neuroblastoma cell lines and two noncancerous cell lines—mesenchymal stem cells (MSCs). **Methods**: The influence of polyphenols on neuroblastoma cells and MSCs were studied via an MTT-assay, cell cycle analysis, and an apoptosis assay (flow cytometry). Chou-Talalay algorithms were used to quantify drug interactions. SeaHorse energy profiling was applied to study energy metabolism. The influence of the compounds on metabolic enzymes and signaling pathways was examined using immunoblotting. Total protein biosynthesis was assessed using o-propargyl-puromycin labeling (flow cytometry). **Results**: While most of the studied polyphenols displayed a more significant inhibitory effect on neuroblastoma cells than on mesenchymal stem cells (MSCs), we found that the combinations of curcumin and quercetin (CQ) and curcumin, quercetin, and resveratrol (CQR) were significantly superior to the individual compounds. These combinations displayed synergistic effects and inhibited the cell cycle while inducing apoptosis. The CQ and CQR combinations effectively suppressed metabolic reprogramming by downregulating key enzymes of glycolysis, respiration, one-carbon metabolism, glutaminolysis, and fatty acid biosynthesis, as well as N-Myc and c-Myc, which are master regulators of metabolic processes. Furthermore, CQ and CQR inhibited AKT/mTOR, MAPK/ERK, and WNT/β-catenin signaling pathways and total protein biosynthesis and sensitized malignant cells to doxorubicin. **Conclusions**: Polyphenol combinations exert multifaceted inhibitory effects on metabolic rewiring and signaling networks in neuroblastoma cells.

## 1. Introduction

Phytochemicals are considered promising candidates for improving treatment efficiency and decreasing adverse effects in cancer patients [1,2,3]. Dietary polyphenols, a group of naturally occurring plant compounds, are among them and have multiple pharmacological activities. They are found in various foods, such as fruits, vegetables, tea, coffee, wine, and cocoa. A high daily intake of dietary polyphenols has been linked to a reduced risk of chronic diseases, such as cardiovascular disease, neurodegenerative illnesses, cancer, and obesity [4,5,6,7].

Due to their abundance and low risk, as well as their variety of health benefits, dietary polyphenols have been the subject of extensive study in a large number of clinical trials aimed at treating various diseases [8]. Some of them are very popular dietary supplements worldwide. Curcumin (beta-diketone), for instance, is a yellow pigment found in turmeric (Curcuma longa) and is also known as the “golden spice”. It has been used for millennia in traditional medicine and is currently the focus of intense scientific research. Another example is quercetin, a flavonoid found in various fruits, vegetables, leaves, seeds, and grains. Resveratrol is a naturally occurring polyphenol (stilbene) found in certain plants, including grapes, some berries, and peanuts. Epigallocatechin gallate (EGCG) is the most abundant catechin in green tea. Kaempferol (3,4,5,7-tetrahydroxyflavone) is found in various vegetables and fruits, such as beans, broccoli, cabbage, strawberries, tea, grapes, and apples.

Neuroblastoma is an aggressive and heterogeneous solid tumor that originates from immature cells or precursors of the sympathetic nervous system during embryonic development or the early postnatal period. It accounts for 8 to 10% of malignancies in patients younger than 15 years of age and is responsible for 15% of pediatric cancer deaths. The disease is often diagnosed in its later stages [9]. Fifty percent of initial cases metastasize to regional lymph nodes, bone marrow, bone, liver, and skin. In children aged 18 months and older, these tumors are often inoperable or metastatic. These cases require intensive multimodality therapy and have a survival rate of less than 50% [10].

Depending on age, stage, and tumor biology, neuroblastomas are classified as low, intermediate, or high risk, which influences treatment methods and prognosis. Aberrant MYCN activity caused by chromosomal MYCN amplification is a primary driver of high-risk neuroblastoma, which has an overall survival rate of less than 50% [11,12].

Several signaling pathways, including PI3K/AKT/mTOR, Notch, Wnt, and MAPK/ERK, are typically deregulated in neuroblastomas [13]. Furthermore, metabolic rewiring, a hallmark of cancer, is prevalent in neuroblastomas, especially in NMYC-amplified tumors [11]. These processes are promising drug targets for adjuvant neuroblastoma therapy.

Conversely, numerous dietary polyphenols have been observed to impede various metabolic and signaling pathways. In addition, the available data suggest that the utilization of certain dietary polyphenols in the treatment of neuroblastoma is a viable approach [14,15].

In this study, we compared the multiple inhibitory activities of selected dietary polyphenols and their combinations on key metabolic and signaling pathways in two neuroblastoma cell lines with and without MYCN amplification.

## 2. Results

### 2.1. Screening Some Dietary Polyphenols for Their Cytotoxic Properties in Human Neuroblastoma and Mesenchymal Stem Cell Lines

First of all, based on literature, we selected a set of common polyphenols that have been studied in clinical trials for various purposes and are widely used as dietary supplements. This set includes curcumin, resveratrol, quercetin, kaempferol, and EGCG [16]. We screened these polyphenols for their cytotoxic and cytostatic properties against two subtypes of human neuroblastoma cell lines: IMR32 (MYCN-amplified) and SH-SY5Y (MYCN non-amplified). To assess the impact of the compounds on noncancerous cells, we used two human mesenchymal stem cell lines, DF2 and FRSN, which were derived from the skin.

**Figure 1:**
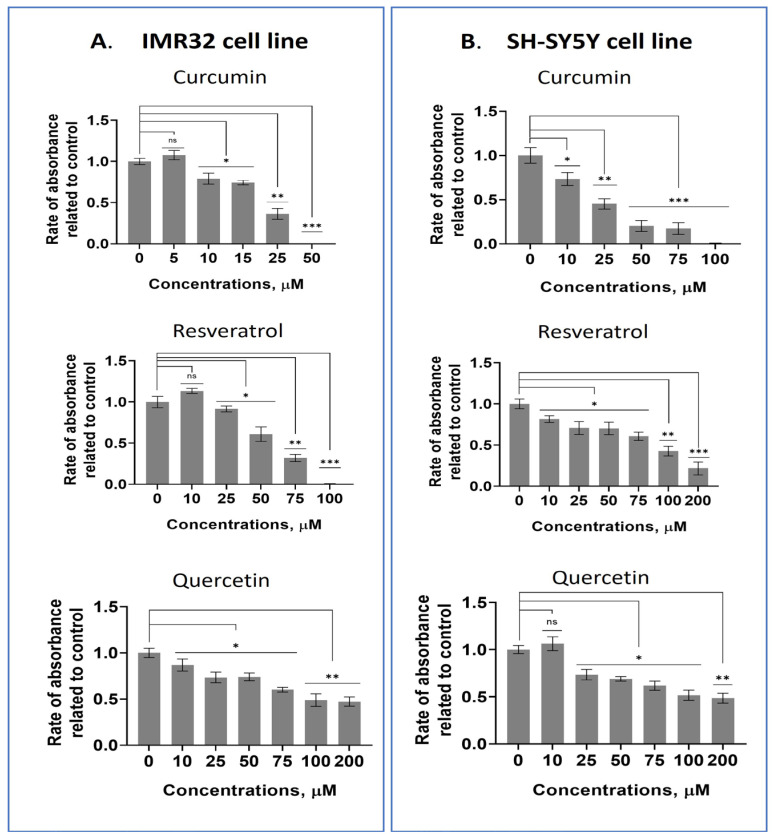
Curcumin, resveratrol, and quercetin suppress neuroblastoma cell lines. (**A**) IMR32 cell line. (**B**) SH-SY5Y cell line. MTT data are shown. A Mann–Whitney U-test was applied to check for statistically significant differences. * *p* ≤ 0.05; ** *p* ≤ 0.01; *** *p* ≤ 0.001; ns—nonsignificant.

We performed an MTT assay to compare the effects of the compounds on neuroblastoma and mesenchymal stem cells. The IC50 values for all the studied compounds are listed in Table 1. Among the tested compounds, curcumin, resveratrol, and quercetin were the most effective at suppressing neuroblastoma cells while being the least toxic to non-cancerous cell lines (Table 1, Figure 1, Figures S1 and S2). Based on these results regarding efficiency and selectivity, curcumin, quercetin, and resveratrol were chosen for further research. It is important to note that the IC50 values for neuroblastoma cells were significantly smaller than those for non-cancerous cells (DF2 and FRSN) for all polyphenols studied. Moreover, all concentrations of kaempferol increased the proliferation of both non-cancerous cell lines, whereas quercetin and resveratrol stimulate the growth of DF2 cells (Figures S1 and S2).

### 2.2. Curcumin, Quercetin, and Resveratrol in Combination Display Synergistic Down-Regulation of Neuroblastoma Cell Lines

Since polyphenols often occur together in our diet, we focused on studying their combined effects. To accomplish this, we treated neuroblastoma cell models with three polyphenols (curcumin, quercetin, and resveratrol) separately and in combination at concentrations of 10 and 25 μM, followed by MTT assay. We processed the obtained data using Chow-Talalau algorithms [17], which allow for the quantitative characterization of drug effects and interactions.

**Figure 2:**
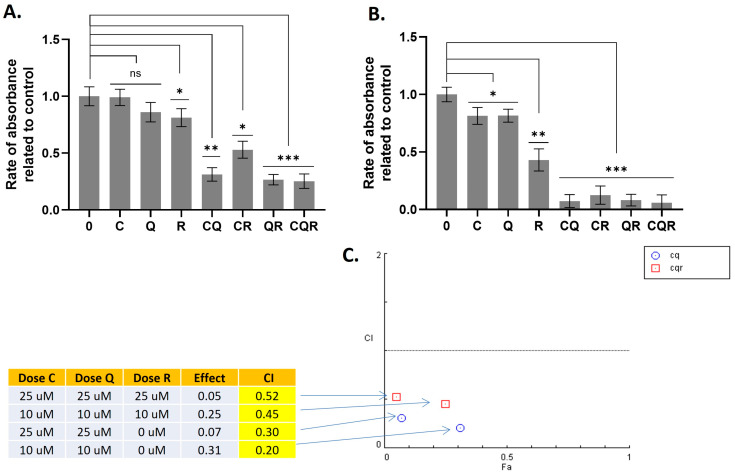
The combinations of polyphenols that synergistically suppress IMR32 cells. Results of the MTT test demonstrating the inhibition of IMR32 cells with (**A**) 10 uM and (**B**) 25 uM of the compounds studied. Results are represented as the mean ± SEM. A Mann–Whitney U-test was applied to check for statistically significant differences. * *p* ≤ 0.05; ** *p* ≤ 0.01; *** *p* ≤ 0.001; ns—nonsignificant. (**C**) Combination Index (CI) plots which reflect the distribution of CIs for different combinations of C/Q/R concentrations depending on their effects (rate of absorbance related to control). CI plots were calculated using Chou-Talalay algorithms (CompuSyn software, version 1.0, http://www.combosyn.com/; access on 12 February 2025). CI < 1 demonstrates the synergistic action of drugs. C—curcumin, Q—quercetin, R—resveratrol, CQ—curcumin with quercetin, CR—curcumin with resveratrol, QR—quercetin with resveratrol, and CQR—curcumin with quercetin and resveratrol.

**Figure 3:**
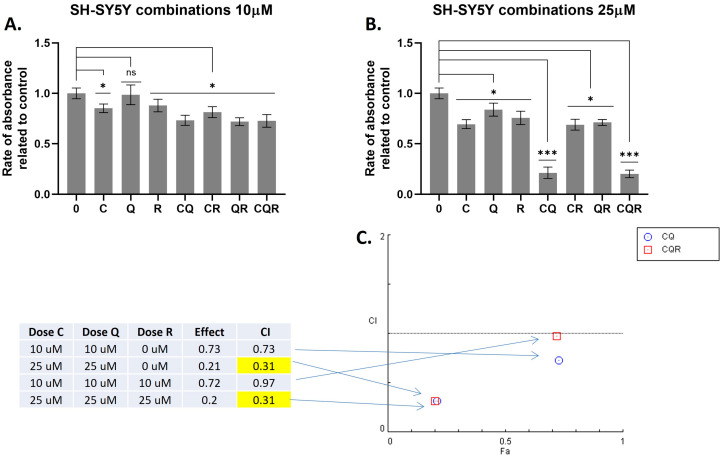
The combinations of polyphenols that synergistically suppress SH-SY5Y cells. Results of the MTT test demonstrating the inhibition of IMR32 cells with (**A**) 10 uM and (**B**) 25 uM of the compounds studied. Results are represented as the mean ± SEM. A Mann–Whitney U-test was applied to check for statistically significant differences. * *p* ≤ 0.05; *** *p* ≤ 0.001; ns—nonsignificant. (**C**) Combination Index (CI) plots which reflect the distribution of CIs for different combinations of C/Q/R concentrations depending on their effects (rate of absorbance related to control). CI plots were calculated using Chou-Talalay algorithms (CompuSyn software, version 1.0, http://www.combosyn.com/, access on 12 February 2025). CI < 1 demonstrates the synergistic action of drugs. C—curcumin, Q—quercetin, R—resveratrol, CQ—curcumin with quercetin, CR—curcumin with resveratrol, QR—quercetin with resveratrol, and CQR—curcumin with quercetin and resveratrol.

The results (Figure 2 and Figure 3) demonstrate that combinations of curcumin with quercetin (CQ), and curcumin with quercetin and resveratrol (CQR) were effective, while other combinations were not. A strong cytotoxic effect was observed for these combinations in both neuroblastoma cell lines, including SH-SY5Y cells, which are quite resistant to various cytotoxic drugs due to their expression of ABC transporters (primarily MRP1), which provide multiple drug resistance [18].

Low values of the combination index (CI < 1) indicate a synergistic effect between CQ and CQR combinations (Figure 2 and Figure 3). The results of the MTT test also show that curcumin primarily contributes to cytotoxicity. The addition of quercetin to curcumin, as well as quercetin combined with resveratrol, significantly increases curcumin’s toxicity. Conversely, adding resveratrol alone to curcumin significantly suppresses its toxicity, indicating an antagonistic interaction (Figure 2 and Figure 3).

Based on these results, we investigated other polyphenol combinations by adding kaempferol and EGCG to the existing combinations. However, as shown in Figure S3A,B, introducing or replacing other polyphenols in the effective combinations CQ and CQR does not significantly increase efficiency; sometimes, it reduces cytotoxicity. Thus, we concluded that CQ and CQR are the best combinations for suppressing the studied neuroblastoma cell models.

We also assessed the effect of these combinations on non-tumor cells (MSCs) at the same concentrations. As shown in Figure S3C,D, the effect of these combinations on mesenchymal stem cells was several times lower than on tumor cells, indicating selectivity.

### 2.3. The Combination of Curcumin, Quercetin, and Resveratrol Arrests the Cell Cycle and Induces Cell Death of Neuroblastoma Cells

As the results of the MTT assay reflect the cytostatic, cytotoxic, and metabolically lowering activity of the compounds, we have further studied the effects of polyphenol combinations in neuroblastoma cells.

**Figure 4:**
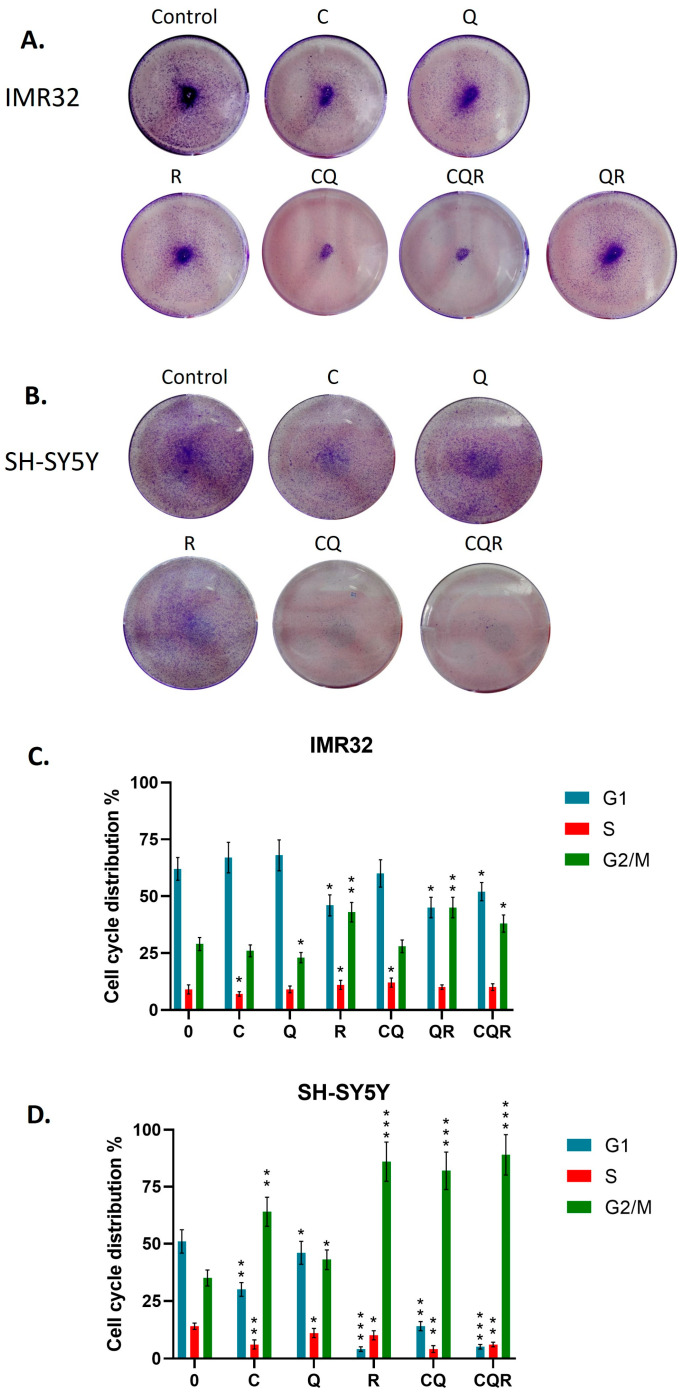
Dietary polyphenols and their combinations inhibit the growth and cell cycle of neuroblastoma cell lines. (**A**) and (**B**) Crystal violet assay and cell cycle assay of (**C**) IMR32 and (**D**) SH-SY5Y cell lines treated with both individual polyphenols and their combinations. Student’s *t*-test was applied to check pairwise statistically significant differences. * *p* ≤ 0.05; ** *p* ≤ 0.01, *** *p* ≤ 0.001. C—curcumin, Q—quercetin, R—resveratrol, CQ—curcumin with quercetin, QR—quercetin with resveratrol, and CQR—curcumin with quercetin and resveratrol.

First of all, we verified the results of the MTT assay by orthogonal methods of cell proliferation and cell cycle analysis. We treated the cells with the studied substances, as well as their combinations, and after 48 h fixed the cells and stained them with crystal violet. Figure 4A,B show that combinations (CQ and CQR) dramatically reduced the number of cells compared to the control and individual compounds, suggesting the cytotoxic effects.

Then, we have compared the impact of polyphenol combinations and individual compounds on the cell cycle. Figure 4C,D show that CQ and CQR combinations induced G2/M cell cycle arrest slightly more than resveratrol alone.

**Figure 5:**
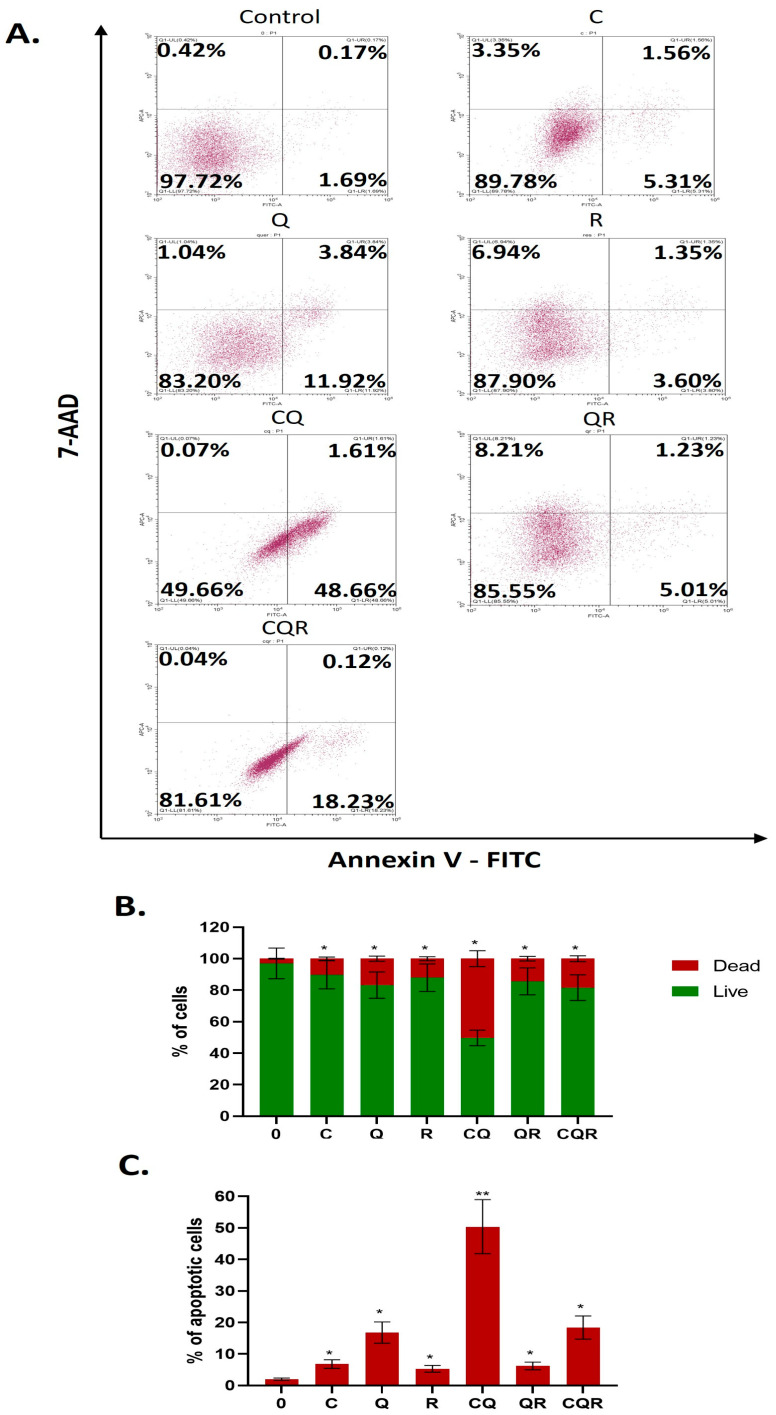
Dietary polyphenols and their combinations induce the apoptosis of SH-SY5Y neuroblastoma cell lines. Results of Annexin V/7-AAD assay. (**A**) Flow cytometry plots. Diagrams illustrate (**B**) the proportion of living/dead cells and (**C**) the percentage of apoptotic cells following treatment with both individual compounds and their combinations. Results are represented as the mean ± SEM. Student’s *t*-test was applied to check pairwise statistically significant differences. * *p* ≤ 0.05; ** *p* ≤ 0.01. C—curcumin, Q—quercetin, R—resveratrol, CQ—curcumin with quercetin, QR—quercetin with resveratrol, and CQR—curcumin with quercetin and resveratrol.

Finally, we have analyzed the cell death using Annexin V-FITC/7-AAD double staining and flow cytometry. The analysis demonstrated that selected combinations of polyphenols (CQ and CQR) significantly increased cell death in neuroblastoma models compared with exposure to individual polyphenols (Figure 5 and Figure S4), which proves their cytotoxic mode of action.

### 2.4. The Polyphenol Combinations Inhibit Energy Metabolism in Neuroblastoma Cells

Metabolic reprogramming is considered to be one of the “hallmarks of cancer” and an important target for therapeutic intervention, including neuroblastoma [11,19]. Additionally, curcumin, resveratrol, and quercetin are known to negatively affect metabolic processes in cancer cells [16]. Therefore, we decided to study the effect of polyphenol combination on metabolic rewiring in neuroblastoma cell models.

To do this, we first used SeaHorse energy profiling technology, which allows us to assess the intensity of glycolysis and respiration. For the analysis, SH-SY5Y and IMR32 cell lines were treated with the studied polyphenols and their combinations for 24 h, after which the SeaHorse ATP real-time synthesis assay kit (Agilent) was applied.

**Figure 6:**
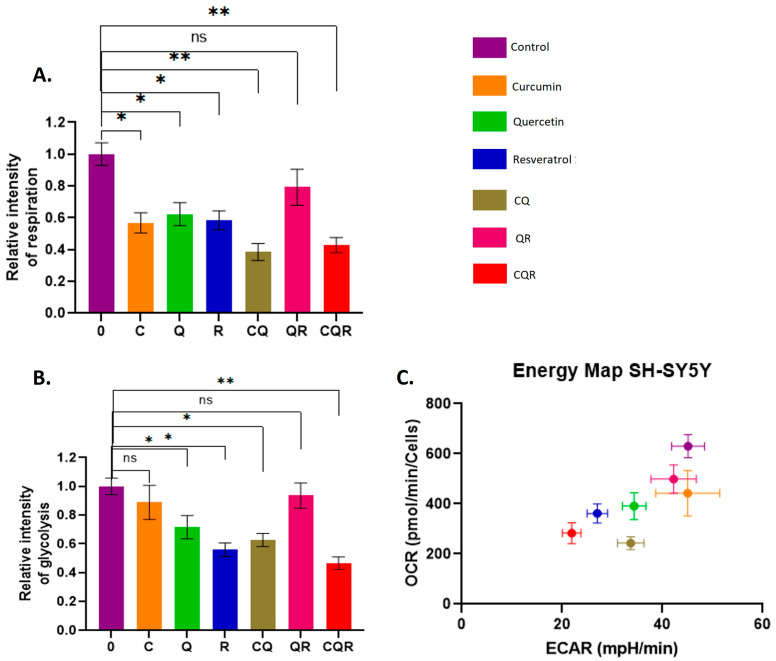
Dietary polyphenols and their combinations suppress glycolysis and respiration in SH-SY5Y cells. SeaHorse Data. The diagrams illustrate the intensity of (**A**) respiration and (**B**) glycolysis. (**C**) The energy diagram reflects the intensity of glycolysis (ECAR) and respiration (OCR). OCR—Oxygen Consumption Rate (shows respiration); ECAR—Extracellular Acidification Rate (shows glycolysis). A Mann–Whitney U-test was applied to check for statistically significant differences. * *p* ≤ 0.05; ** *p* ≤ 0.01; ns—nonsignificant. C—curcumin, Q—quercetin, R—resveratrol, CQ—curcumin with quercetin, QR—quercetin with resveratrol, and CQR—curcumin with quercetin and resveratrol.

**Figure 7:**
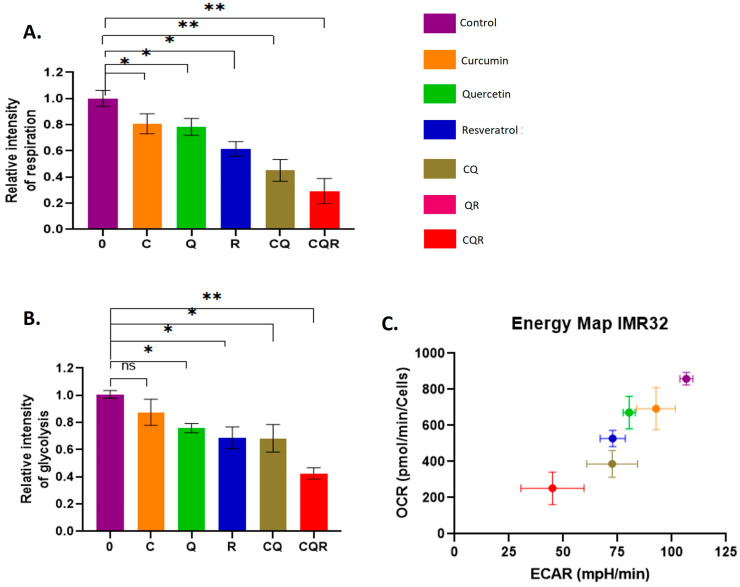
Dietary polyphenols and their combinations suppress glycolysis and respiration in IMR32 cells. SeaHorse Data. The diagrams illustrate the level of (**A**) respiration and (**B**) glycolysis. (**C**) The energy diagram reflects the intensity of glycolysis (ECAR) and respiration (OCR). OCR—Oxygen Consumption Rate (shows respiration); ECAR—Extracellular Acidification Rate (shows glycolysis). A Mann–Whitney U-test was applied to check for statistically significant differences. *p ≤ 0.05; ** *p* ≤ 0.01; ns—nonsignificant. C—curcumin, Q—quercetin, R—resveratrol, CQ—curcumin with quercetin, and CQR—curcumin with quercetin and resveratrol.

In both cell lines, all three compounds we studied (except for curcumin in the SH-SY-5Y line) reduced both glycolysis and respiration (Figure 6 and Figure 7). At the same time, the inhibitory effect of the polyphenol combinations, especially CQR, was much stronger compared to the effect of individual substances. The exception was the QR combination, which showed an antagonistic effect, i.e., an increase in the intensity of glycolysis and respiration compared to the effect of individual compounds. However, these results match the MTT test data perfectly and suggest that polyphenol combinations (mainly CQR) could be effective in reducing the energy metabolism of neuroblastoma cells.

### 2.5. The Polyphenol Combinations Have Multiple Inhibitory Impacts on Metabolic Rewiring in Neuroblastoma Cells

Metabolic rewiring is a complex process including both the plasticity of metabolic regulation and alterations of a number of important metabolic pathways—glycolysis, respiration, one-carbon and glutamine metabolism, de novo lipogenesis, etc.

To study polyphenols and their combinations on the above-mentioned metabolic processes, we used Western blot for key enzymes of onco-associated metabolic processes: HK2, PFKM, PKM2, LDHA (glycolysis), FASN (de novo lipogenesis), GLS1 (glutaminolysis), PSAT1, and SHMT2 (enzymes of one-carbon metabolism-serine biosynthesis and folate cycle); as well as for driver oncogenes—c-Myc and N-Myc, which are key transcriptional regulators of the studied enzymes.

**Figure 8:**
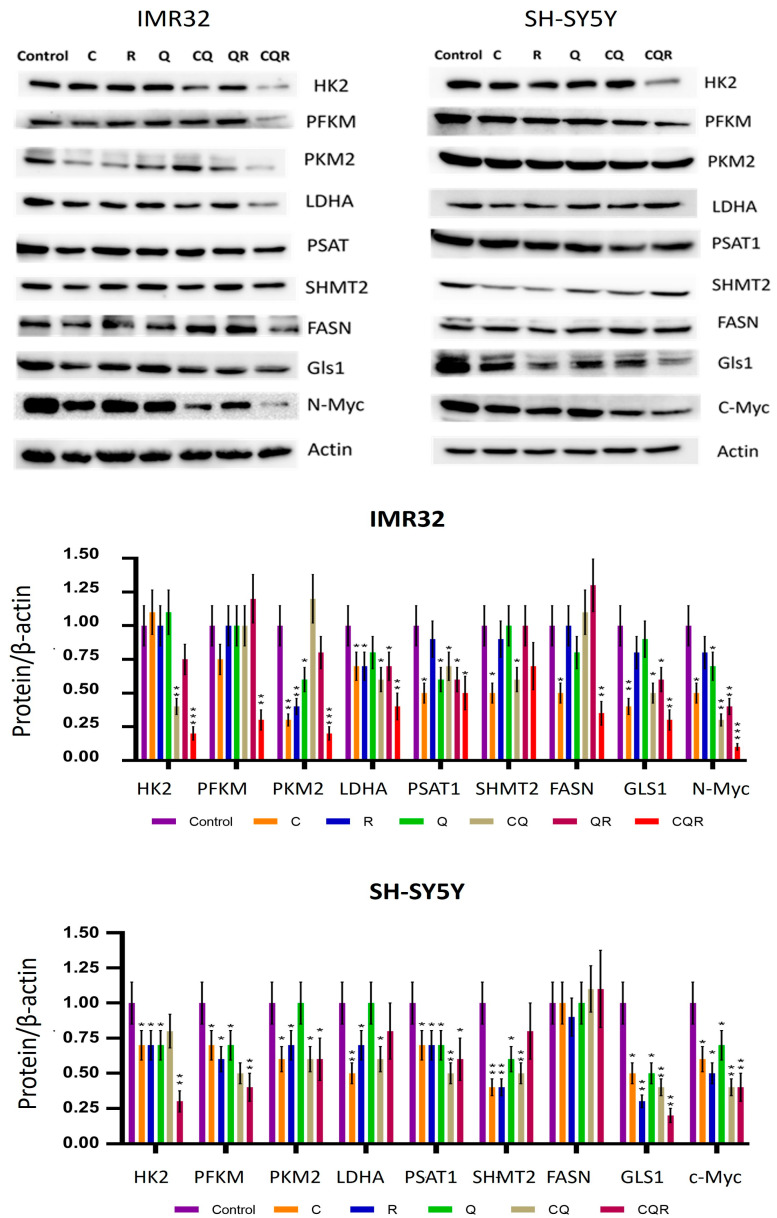
Polyphenols and their combinations suppress the key enzymes involved with glycolysis (HK2, PFKM, PKM2, LDHA), one-carbon metabolism (PSAT1, SHMT2), de novo fatty acids biosynthesis (FASN), glutaminolysis (GLS1), and their important transcriptional regulators (N-Myc, c-Myc) at the protein level (Western blot) in IMR32 and SH-SY5Y neuroblastoma cell lines. The diagrams show the results of quantification performed using Image J software (Version 1.54p). Student’s *t*-test was used to determine pairwise statistically significant differences. Asterisks represent a significant difference in protein expression (* *p* ≤ 0.05; ** *p* ≤ 0.01; *** *p* ≤ 0.001). C—curcumin, Q—quercetin, R—resveratrol, CQ—curcumin with quercetin, QR—quercetin with resveratrol, and CQR—curcumin with quercetin and resveratrol.

As seen from Figure 8, individual polyphenols suppress the expression of some of these enzymes. However, their combinations, especially CQR, significantly suppress almost all the studied key enzymes of the main onco-associated metabolic pathways, as well as their most important transcriptional regulators—c-Myc and N-Myc. That is, the combination of CQR polyphenols (curcumin, quercetin, and resveratrol) identified in this work demonstrated multitargeting in relation to various metabolic processes in neuroblastomas.

### 2.6. The Polyphenol Combinations Inhibit Different Signaling Pathways in Neuroblastoma Cell Lines

Oncogenesis in general, and metabolic rewiring in particular, are tightly linked to deregulated signaling pathways. To assess the impact of polyphenols and their combinations on the signaling in neuroblastoma cell lines, we have applied Western blot for the key components of AKT/mTOR, MAPK/ERK, and β-catenin signaling pathways that are frequently altered in neuroblastoma.

**Figure 9:**
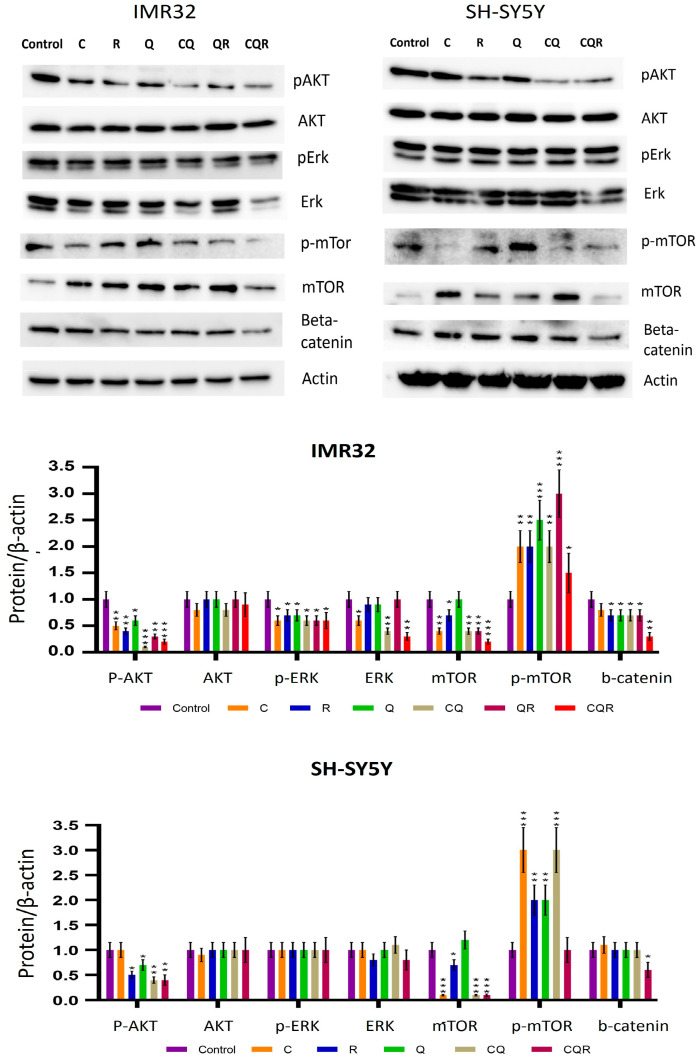
Polyphenols and their combinations inhibit AKT/mTOR, MAPK/ERK, and Wnt/β-catenin signaling pathways in neuroblastoma cell lines. Western blotting was carried out. The diagrams show the results of quantification performed using Image J software (Version 1.54p). Student’s *t*-test was used to determine pairwise statistically significant differences. Asterisks represent a significant difference in protein expression (* *p* ≤ 0.05; ** *p* ≤ 0.01; *** *p* ≤ 0.001). C—curcumin, Q—quercetin, R—resveratrol, CQ—curcumin with quercetin, QR—quercetin with resveratrol, and CQR—curcumin with quercetin and resveratrol.

The results in Figure 9 demonstrate that generally individual polyphenols down-regulate the signaling proteins studied. However, the polyphenol combinations (CQ and CQR) display multiple inhibitory impacts on signaling pathways, with the exception of the MAPK/ERK pathway in SH-SY5Y cells, suggesting they may be used to target both metabolic and signaling networks in neuroblastomas.

### 2.7. The Polyphenol Combinations Suppress Total Protein Biosynthesis in Neuroblastoma Cell Lines

The PI3K/AKT/mTOR signaling pathway is tightly linked to anabolism, as mTOR is the key regulator of protein biosynthesis [20]. Thus, we have questioned if CQ and CQR-mediated inhibition of p-mTOR (the active form of mTOR protein) results in the down-regulation of protein biosynthesis in neuroblastoma cells.

**Figure 10:**
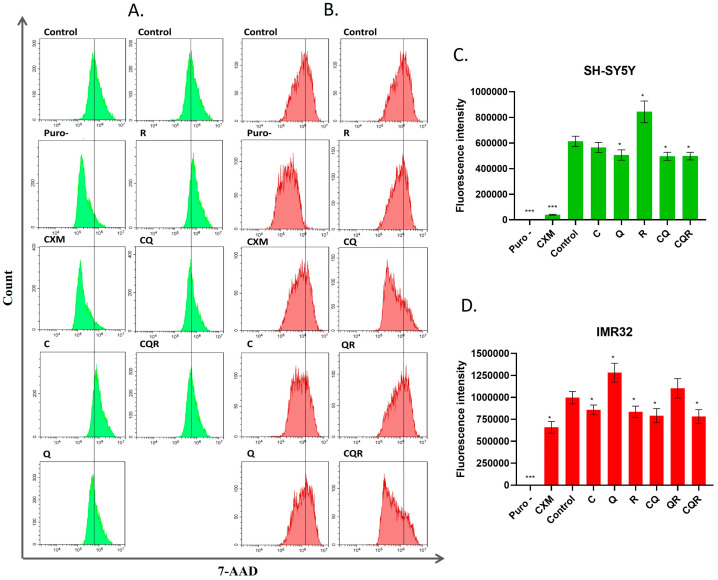
Polyphenols (10 μM) and their combinations (10 μM of each) inhibit total protein biosynthesis in neuroblastoma cell lines. Flow cytometry plots of propargyl-puromycin—labeled (**A**) SH-SY5Y and (**B**) IMR32 cell lines pretreated with individual polyphenols and their combinations for 24 h. ‘Medians’ of the peak for control samples are showed by vertical bar. (**C**,**D**) Diagrams showing the intensity of total protein biosynthesis in SH-SY5Y and IMR32 cells, respectively, pretreated with either 10 μM of individual polyphenols and their combinations. The ‘mean’ values of fluorescence intensities have been used for calculations. The values for control cells that were not labeled with o-propargyl-puromycin (puro) have been subtracted from the values for each sample. Results are represented as mean ± SEM. Student’s *t*-test was used to determine pairwise statistically significant differences. Asterisks represent a significant difference in fluorescence intensity related to control (* *p* ≤ 0.05; *** *p* ≤ 0.001). C—curcumin, Q—quercetin, R—resveratrol, CQ—curcumin with quercetin, QR—quercetin with resveratrol, CQR—curcumin with quercetin and resveratrol.

To do this, we treated IMR32 and SH-SY5Y cell lines with polyphenols and their combinations and applied a total protein biosynthesis kit based on the o-propargyl puromycin inclusion in the synthetizing proteins that is detected by flow cytometry. According to the results shown in Figure 10, CQ and CQR combinations effectively inhibited total protein biosynthesis, which aligns with their inhibitory activity on energy metabolism, metabolic enzymes, and the AKT/mTOR signaling pathways.

### 2.8. Polyphenols and Their Combinations Increase the Susceptibility of SH-SY5Y to Doxorubicin

Doxorubicin is a common part of genotoxic therapy for treating neuroblastoma. It is important to note that curcumin, quercetin, and resveratrol have been shown to make cancer cells more sensitive to this treatment [21,22,23].

Thus, we asked if polyphenol combinations could improve the sensitivity of neuroblastoma cells to doxorubicin. As our previous study uncovered that the susceptibility to doxorubicin of IMR32 and SH-SY5Y cells differs by more than 10 times, we tried to sensitize doxorubicin-resistant SH-SY5Y cells by f individual polyphenols and their combination. To do this, we carried out an MTT assay and analyzed the rate of cell death by Annexin V/7-AAD double staining followed by flow cytometry.

**Figure 11:**
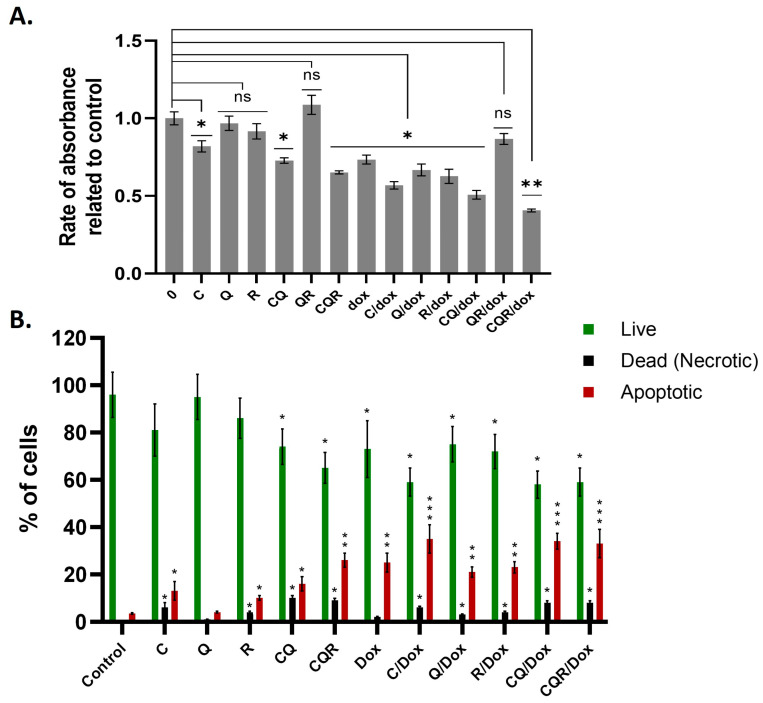
Dietary polyphenols and their combinations sensitize SH-SY5Y neuroblastoma cells to doxorubicin. Cells were treated for 48 h with 10 μM of individual polyphenols, their combinations (each of 10 μM), and 0.5 μM of doxorubicin. (**A**) MTT data. (**B**) Annexin V/7-AAD assay data. Flow cytometry plots are depicted in Figure S5. A Mann–Whitney U-test was performed to detect statistically significant differences in the MTT assay, and a Student’s *t*-test was performed to detect statistically significant differences in the Annexin V/7-AAD assay. Asterisks represent a significant difference in absorbance relative to control (* *p* ≤ 0.05; ** *p* ≤ 0.01; *** *p* ≤ 0.001; ns—nonsignificant). C—curcumin, Q—quercetin, R—resveratrol, CQ—curcumin with quercetin, CQR—curcumin with quercetin and resveratrol, Dox—doxorubicin, C/Dox—curcumin with doxorubicin, Q/Dox—quercetin with doxorubicin, R/Dox—resveratrol with doxorubicin, CQ/Dox—curcumin with quercetin and doxorubicin, CQR/Dox—curcumin with quercetin, resveratrol, and doxorubicin.

The results in Figure 11A,B show that all three polyphenols we tested made SH-SY5Y cells more sensitive to doxorubicin, and the combinations of CQ and especially CQR greatly increased their sensitivity, leading to a big drop in absorbance in the MTT test and a higher percentage of dead and apoptotic cells in the Annexin V/7-AAD test.

## 3. Discussion

Many polyphenols are abundant in nature and our diet and display multiple pharmacological activities, including antioxidant, anti-aging, neuroprotective, etc. [24]. They can also affect the immune system and might help prevent and treat autoimmune diseases like type 1 diabetes, rheumatoid arthritis, and multiple sclerosis by changing signaling pathways, reducing inflammation, and restricting demyelination [25,26]. Different studies have also revealed anticancer properties of polyphenols [16,27,28].

In the present study, we screened some polyphenols, which are abundant in our diet, to suppress human neuroblastoma cell lines. Notwithstanding the finding that all polyphenols exhibited a more substantial inhibitory effect on neuroblastoma than on mesenchymal stem cell lines (non-cancerous cells), the combination of curcumin with quercetin (CQ) and curcumin with quercetin and resveratrol (CQR) was found to be significantly more potent than the individual compounds. These combinations exhibited synergistic effects in suppressing neuroblastoma cell lines, inhibiting the cell cycle, and inducing apoptosis.

Importantly, CQ and CQR exhibited a substantial inhibitory effect on metabolic reprogramming. This effect was achieved through the suppression of pivotal metabolic enzymes involved in multiple oncogenic metabolic pathways, including glycolysis, respiration, one-carbon metabolism, glutaminolysis, and fatty acid biosynthesis. Furthermore, these combinations significantly suppressed AKT/mTOR, ERK, and β-catenin–mediated signaling pathways that are critical for neuroblastoma development. In addition, the polyphenol combinations demonstrated the ability to suppress N-Myc, the oncogene driver implicated in neuroblastoma, as well as its paralog, c-Myc.

Metabolic reprogramming has been identified as a hallmark of various malignancies [29,30,31], including neuroblastoma, and is an essential component of the c-Myc’s and N-MYC’s growth-promoting program, which stimulates cell growth and proliferation by enhancing glucose and glutamine uptake, energy production, and macromolecule biosynthesis [11,32,33,34]. Importantly, our previous data revealed the mutual exclusion of either N-Myc or C-Myc expression in different neuroblastoma samples [35] implying polyphenol combinations mediated the suppression of metabolic rewiring in both NMYC amplified and non-amplified neuroblastomas.

Finally, the polyphenol combinations CQ and CQR compromised total protein biosynthesis, indicating an inhibitory impact on anabolic processes. These data are in accordance with the inhibitory effect of CQ and CQR on p-AKT and p-mTOR levels. The PI3K/Akt/mTOR pathway is an important signaling cascade that regulates cell growth and different anabolic processes, including protein synthesis [20]. The activation of Akt in turn usually augments the activity of mTOR and the latter stimulates different anabolic processes, primarily protein synthesis [36,37].

In accordance with these findings, CQ and CQR have been observed to suppress the energy metabolism (glycolysis and respiration), the protein levels of FASN (a key enzyme in fatty acid biosynthesis), PSAT1, and SHMT2 (key enzymes in serine biosynthesis and one-carbon metabolism). Beyond its role in ATP production, glycolysis facilitates anabolic processes through its intermediates, which serve as precursors of amino acids, ribose, and lipids [38,39]. Additionally, glycolysis has been shown to support one-carbon metabolism, an important process that provides nucleotides and helps keep redox and epigenetic balance, both of which are essential for the growth of cancer cells [16,40]. According to numerous literature data, all of the metabolic pathways studied (glycolysis, glutaminolysis, fatty acid biosynthesis, and one-carbon metabolism), as well as mTOR and NMYC, are important and prospective drug targets in neuroblastoma [11,41,42,43,44]. Consequently, the CQ and CQR combinations have been demonstrated to exert a comprehensive inhibitory effect on both catabolic (i.e., the energy metabolism and glutaminolysis) and anabolic processes (i.e., protein and fatty acid biosynthesis and one-carbon metabolism). This observation suggests that these combinations may serve as promising adjuvants in the treatment of neuroblastoma.

Different molecular mechanisms may underlie the observed effects. Kafoud with colleagues [14] summarized the mechanisms by which various polyphenols, including curcumin, quercetin, and resveratrol, suppress neuroblastoma cell and animal models through anti-proliferative and apoptotic mechanisms. Curcumin, quercetin, and resveratrol are small molecules and are known to interact with a wide array of proteins through various mechanisms, including physical binding and indirect modulation of signaling pathways [45,46,47,48,49]. For instance, curcumin down-regulates PI3K/Akt, MAPK, and Wnt/β-catenin signaling pathways; inhibits glycolysis, respiration, glutaminolysis, and de novo lipogenesis [50,51,52,53].

Resveratrol has been demonstrated to exert its biological effects by impeding the Akt and Erk signaling pathways, thereby suppressing the expression of HK2, PKM2, and c-Myc. In addition, it has been shown to inhibit glycolysis and oxidative phosphorylation in various cancer cell models and murine models [54,55,56,57]. In turn, quercetin has been shown to inhibit PI3K/Akt/mTOR-, MAPK/ERK-, and NF-κB-dependent signaling. It has also been demonstrated to suppress HK2, PKM2, LDHA, FASN, mitigating glycolysis, oxidative phosphorylation, and fatty acids biosynthesis [58,59,60,61,62].

Furthermore, all three polyphenols have been demonstrated to bind G-quadruplex structures in the c-Myc promoter, thereby inhibiting its expression [63,64].

It is noteworthy that our data indicate the suppression of neuroblastoma cells by individual polyphenols (i.e., curcumin, quercetin, and resveratrol). These polyphenols also inhibit metabolic and signaling pathways to a certain extent. However, their inhibitory activities vary between the specific cell lines and processes that have been studied. However, CQ and, particularly, CQR demonstrated the most efficient inhibition of all the biochemical processes studied in both cell lines. This suggests that these combinations possess multiple inhibitory activities in respect to metabolic and signaling pathways in neuroblastoma.

Curcumin, quercetin, and resveratrol have all been used in clinical trials to treat a number of diseases, including different types of malignancies [65,66,67]. For example, curcumin has been tested in cancer-related clinical trials 22 times, shown to be safe and effective, and helped reduce the side effects of standard treatments [68,69,70].

Importantly, cancer chemopreventive activity against various malignancies was also shown for curcumin, quercetin, and resveratrol [7,58,71,72,73,74]. It has been suggested that a polyphenol-rich diet, potentially surpassing 650 milligrams per day, might be advantageous to overall health and reduced mortality [75,76,77]. Moreover, there is evidence that such a diet may reduce some types of cancer [78,79]. Collectively, the results of our present study provide additional molecular insights into the mechanism of cancer chemoprevention by polyphenols with a particular focus on neuroblastoma cells.

## 4. Materials and Methods

### 4.1. Cell Lines and Reagents

Neuroblastoma cell lines used in this study (IMR32 and SH-SY5Y) were purchased from ATCC. MSC lines (DF2 and FRSN) were obtained from the shared research facility “Vertebrate cell culture collection” (INC RAS). All cells were grown in DMEM media supplemented with 10% fetal bovine serum, 100 μg/mL gentamycin, and 2 mM l-glutamine at 37 °C in 5% CO_2_ atmosphere.

Curcumin (Shanghai Macklin Biochemical Technology Co., Shanghai, China, 98% purity), quercetin (Shanghai Macklin Biochemical Technology Co., Shanghai, China, 97% purity), resveratrol (Shanghai Macklin Biochemical Technology Co., Shanghai, China, 99% purity), kaempferol (Shanghai Macklin Biochemical Technology Co., Shanghai, China, 97% purity), EGCG (epigallocatechin gallate) (Shanghai Macklin Biochemical Technology Co., Shanghai, China, 98% purity) were dissolved in DMSO. All experiments with 20E used DMSO as a control.

### 4.2. MTT Assay

The day before treatment, 2500 cells were seeded per well in a 96-well plate. After a 24 h incubation period, the cells were exposed to polyphenols at various concentrations for an additional 48 h. DMSO was used as a control. On the day of analysis, 10 μL of a 5 mg/mL thiazolyl blue solution (Paneko, Moscow, Russia) was added to each well. The plates were then incubated for two hours at 37 °C in a CO_2_ incubator. Then, the thiazolyl blue-containing medium was discarded, and 100 μL of DMSO was added to dissolve the MTT formazan salt. Absorbances at 570 and 630 nm (reference) were measured using a Bio-Rad iMark microplate reader (Bio-Rad, Hercules, CA, USA). The results are presented as the mean ± SEM. IC50 was calculated using the AAT Bioquest IC50 online calculator (https://www.aatbio.com/tools/ic50-calculator/; access on 10 February 2025).

### 4.3. Assessment of Drug Synergy

IC50 values and drug synergy were evaluated using MTT assay results. Drug interaction assessment was performed using Chou and Talalay algorithms via the CompuSyn software platform (http://www.combosyn.com; access on 12 February 2025, version 1.0 [17]) as described previously [80] The findings were displayed in the form of Combination Index (CI) plots and a table that included values for CI and Dose Reduction Index (DRI). A CI of less than 1 indicates a synergistic interaction between drugs.

### 4.4. Crystal Violet Assay

Cells were seeded at a density of 20,000 cells per well in a 12-well plate. The next day, polyphenols were added to the wells and left for 48 h. DMSO was used as a control. On the day of the study, the medium was removed, and the cells were fixed with 1 mL of 4% paraformaldehyde (PFA). Then, the cells were stained with 1 mL of a solution containing 0.5% crystal violet, 10% methanol, and 1% chloroform.

### 4.5. Western Blot

1,000,000 cells were seeded in 10 cm cell culture dishes. The next day, polyphenols were added for 48 h. On the day of analysis, the cells were lysed using a RIPA buffer containing 150 mM NaCl, 50 mM Tris-HCl (pH 7.5), 0.5% NP-40, 1 mM PMSF, and a protease inhibitor cocktail. Total protein concentration was assessed quantitatively using a BCA assay (Thermo Fisher Scientific, Waltham, MA, USA) and was then diluted with Laemmli buffer. Then, 30 µg of protein lysate samples were analyzed using 10% SDS-PAGE with a Tris-Glycine running buffer and transferred to a PVDF membrane (Bio-Rad, Hercules, CA, USA). The membranes were immersed in a solution of PBS with 5% nonfat milk for one hour for blocking, followed by an overnight incubation with primary antibodies. The current study employed the following antibodies: HK2 (MA5-14849, Thermo Fisher Scientific, Waltham, MA, USA) and PFKM (Affinity Bioscience, DF7362, Wuhan, China), PKM2 (Servicebio, GB11392-100, Wuhan, China), LDHA (GB11342, ServiceBio, Wuhan, China), PSAT1 (Cloud-Clone, PAD861Hu01, Wuhan, China), SHMT2 (DF6347, Cloud-Clone, Wuhan, China), FASN (MA5-14887, Thermo Fisher Scientific, Waltham, USA), Gls1 (PAJ026Hu01, Cloude-Clone, Wuhan, China), N-Myc (MABE333, Sigma-Aldrich, MO, USA), C-Myc (D84C12, Cell Signaling, Danvers, MA, USA) p-AKT (#2971; Cell Signaling, Danvers, MA, USA), AKT (#9272; Cell Signaling, Danvers, MA, USA), p-ERK (#9101; Cell Signaling, Danvers, MA, USA), ERK (#9102, Cell Signaling, Ma, USA), p-mTOR (#2971; Cell Signaling, Danvers, MA, USA), mTOR (ab25580; Abcam, Waltham, MA, USA), β-catenin (sc-7963, Santa-Cruze, CA, USA), and actin (#8457, Cell Signaling, Danvers, MA, USA). After several washes with PBST, secondary anti-mouse or anti-rabbit antibodies conjugated to horseradish peroxidase (1:10,000; Sigma-Aldrich, MO, USA) were administered for one to two hours. The ECL system (Thermo Scientific, Waltham, MA, USA) and ChemiDoc™ Touch Imager (Bio-Rad, Hercules, CA, USA) were used for detection.

### 4.6. SeaHorse Energy Profiling

The Seahorse XFe24 Analyzer (Agilent, Santa Clara, CA, USA) and the Seahorse XF Real-Time ATP Rate Assay Kit (Agilent, Santa Clara, CA, USA) were used to perform energy profiling, following the manufacturer’s instructions. The cells were seeded in 24-well Seahorse plates one day before treatment with the compounds. Polyphenols and their combinations were added to the wells for 48 h. For the analysis, the SeaHorse XF base medium was supplemented with 1 mM pyruvate, 2 mM glutamine, and 10 mM glucose, and the pH was adjusted to 7.4. A stressor mix composed of oligomycin and rotenone/antimycin A (both from Agilent Technologies, Santa Clara, CA, USA) was used to achieve final concentrations of 3 μM of each substance in the wells. The results are presented as the mean ± SEM.

### 4.7. Cell Cycle Analysis

80,000 cells were seeded in 12-well plates. The next day, substances were added to each well and left for 24 h. DMSO was used as a control. On the day of the study, the cells were trypsinized, then washed with PBS. The cells were fixed with 70% alcohol and incubated at −20 °C for one hour. For DNA content staining, the cells were then incubated for 30 min in a 25 μg/mL 7-AAD solution with 1 mg/mL RNase A (Thermo Fisher Scientific, Waltham, MA, USA). The samples were analyzed using a CytoFLEX (Beckman Coulter, Carlsbad, CA, USA) flow cytometer with an APC-A channel (10,000 events have been analyzed). The results were processed using CytExpert software version 2.4 (Beckman Coulter, CA, USA) and presented as the mean ± SEM.

### 4.8. Annexin V Test

To investigate the effect of polyphenols on cell death, we applied annexin V-FITC/7AAD (Thermo Fisher, Waltham, MA, USA) in accordance with the manufacturer’s protocol. 80,000 cells were seeded in 12-well plates. The next day, either polyphenols or their combinations were added for 48 h. Then, cells were trypsinized, washed, and treated with 7AAD and Annexin V-FITC. Results were evaluated using a CytoFLEX flow cytometer (Beckman Coulter, CA, USA), employing FITC and APC-A channels. Median values were used for calculation. The results were processed using CytExpert software version 2.4 (Beckman Coulter, CA, USA) and represented as mean ± SEM.

### 4.9. Total Protein Biosynthesis Assay

80,000 cells were seeded in 12-well plates. The next day, substances were added for 24 h. On the day of the study, O-propargyl puromycin was added to the cells to label newly synthesized proteins. The cells were then incubated for one hour at 37 °C in a CO_2_ incubator. The cells were then washed with a 0.5% bovine serum albumin (BSA) solution in phosphate-buffered saline (PBS) and fixed with 4% formaldehyde. The cells were then incubated in permeabilizing buffer for 15 min. Next, a staining solution containing 150 mM Tris-HCl (pH 8.0), 1 mM CuSO_4_, 500 mM sodium ascorbate, and 0.1 μM Alexa Fluor 647 azide was added, and the cells were incubated for an additional 30 min. The samples were analyzed using a CytoFLEX (Beckman Coulter, Carlsbad, CA, USA) flow cytometer with an APC-A channel. The results were processed using CytExpert software (version 2.4, Beckman Coulter, CA, USA).

### 4.10. Statistical Analysis

We employed Student’s *t*-test to identify pairwise statistically significant differences in experiments with *n* = 3. The Mann–Whitney U-test was utilized for the SeaHorse energy profiling and MTT assays with sample sizes *n* = 5 and *n* = 6, respectively. The analysis was conducted using GraphPad Prism version 8. A statistical difference was established at *p* < 0.05. * *p* < 0.05; ** *p* < 0.01; *** *p* < 0.001; nonsignificant, ns.

## 5. Conclusions

The polyphenol combinations have been demonstrated to exert a multifaceted inhibitory effect on metabolic rewiring and signaling networks in neuroblastoma cells.

## Figures and Tables

**Table 1:** IC50 values for polyphenols studied in neuroblastoma (SH-SY5Y, IMR32) and non-cancerous cell lines (mesenchymal stem cells—DF2, FRSN).

Cell line	Substance/IC50 (µM)				
	Curcumin	Quercetin	Resveratrol	EGCG	Kaempferol
IMR32	19.7 ± 2.2	140.5 ± 12.1	54.6 ± 4.8	43.7 ± 5.1	>200
SH-SY5Y	20.8 ± 2.4	121 ± 9.9	94.2 ± 10.4	182 ± 17.3	167 ± 15.6
DF2	54.6 ± 4.9	>200	151 ± 16.1	151 ± 14.2	>200
FRSN	79.5 ± 8.7	>200	>200	>200	>200

## Data Availability

The original contributions presented in this study are included in the article/Supplementary Material. Further inquiries can be directed to the corresponding author.

## Supplementary Materials

The following supporting information can be downloaded at: https://www.mdpi.com/article/10.3390/ph18111717/s1, Figure S1: The impact of curcumin, resveratrol, and quercetin on human MSC cell lines; Figure S2: The impact of EGCG and kaempferol on (A) human neuroblastoma and noncancerous (MSC) (B) cell lines; Figure S3: The effect of (A) 10 μM and (B) 25 μM expanded polyphenol combinations on SH-SY5Y cells, as well as the effect of individual polyphenols and their combinations on (C) DF2 and (D) FRSN cell lines; Figure S4: Dietary polyphenols and their combinations induce the cell death of IMR32 neuroblastoma cells; Figure S5: Dietary polyphenols and their combinations sensitize SH-SY5Y neuroblastoma cells to doxorubicin.

## Abbreviations

The following abbreviations are used in this manuscript:

C	Curcumin
Q	Quercetin
EGCG	Epigallocatechin gallate
R	Resveratrol
CQ	Curcumin and Quercetin
CQR	Curcumin, Quercetin, and Resveratrol

## References

[1] Kirdeeva Y., Fedorova O., Daks A., Barlev N., Shuvalov O. (2022). How should the worldwide knowledge of traditional cancer healing be integrated with herbs and mushrooms into modern molecular pharmacology?. Pharmaceuticals.

[2] Choudhari A.S., Mandave P.C., Deshpande M., Ranjekar P., Prakash O. (2020). Phytochemicals in cancer treatment: From preclinical studies to clinical practice. Front. Pharmacol..

[3] Fefilova E., Kirdeeva Y., Parfenyev S., Daks A., Fedorova O., Sorokina M., Ha N.X., Huong T.T., Loc V.T., Hai P.T. (2025). MDM2 up-regulates the energy metabolism in NSCLC in a p53-independent manner. Biochem. Biophys. Res. Commun..

[4] Kiyimba T., Yiga P., Bamuwamye M., Ogwok P., Van der Schueren B., Matthys C. (2023). Efficacy of dietary polyphenols from whole foods and purified food polyphenol extracts in optimizing cardiometabolic health: A meta-analysis of randomized controlled trials. Adv. Nutr..

[5] Dzah C.S., Asante-Donyinah D., Letsyo E., Dzikunoo J., Adams Z.S. (2023). Dietary polyphenols and obesity: A review of polyphenol effects on lipid and glucose metabolism, mitochondrial homeostasis, and starch digestibility and absorption. Plant Foods Hum. Nutr..

[6] Da Porto A., Cavarape A., Colussi G., Casarsa V., Catena C., Sechi L.A. (2021). Polyphenols rich diets and risk of type 2 diabetes. Nutrients.

[7] Ding S., Xu S., Fang J., Jiang H. (2020). The protective effect of polyphenols for colorectal cancer. Front. Immunol..

[8] Marino M., Del Bo’ C., Martini D., Porrini M., Riso P. (2020). A review of registered clinical trials on dietary (poly) phenols: Past efforts and possible future directions. Foods.

[9] Johnsen J.I., Dyberg C., Wickström M. (2019). Neuroblastoma—A neural crest derived embryonal malignancy. Front. Mol. Neurosci..

[10] Qiu B., Matthay K.K. (2022). Advancing therapy for neuroblastoma. Nat. Rev. Clin. Oncol..

[11] Bansal M., Gupta A., Ding H.-F. (2022). MYCN and metabolic reprogramming in neuroblastoma. Cancers.

[12] Otte J., Dyberg C., Pepich A., Johnsen J.I. (2021). MYCN function in neuroblastoma development. Front. Oncol..

[13] Benchia D., Bîcă O.D., Sârbu I., Savu B., Farcaș D., Miron I., Postolache A.L., Cojocaru E., Abbo O., Ciongradi C.I. (2025). Targeting Pathways in Neuroblastoma: Advances in Treatment Strategies and Clinical Outcomes. Int. J. Mol. Sci..

[14] Kafoud A., Salahuddin Z., Ibrahim R.S., Al-Janahi R., Mazurakova A., Kubatka P., Büsselberg D. (2023). Potential treatment options for neuroblastoma with polyphenols through anti-proliferative and apoptotic mechanisms. Biomolecules.

[15] Leis K., Baska A., Bereźnicka W., Marjańska A., Mazur E., Lewandowski B.T., Kałużny K., Gałązka P. (2020). Resveratrol in the treatment of neuroblastoma: A review. Rev. Neurosci..

[16] Shuvalov O., Kirdeeva Y., Daks A., Fedorova O., Parfenyev S., Simon H.-U., Barlev N.A. (2023). Phytochemicals target multiple metabolic pathways in cancer. Antioxidants.

[17] Chou T.-C. (2010). Drug combination studies and their synergy quantification using the Chou-Talalay method. Cancer Res..

[18] Corich L., Aranda A., Carrassa L., Bellarosa C., Ostrow J.D., Tiribelli C. (2009). The cytotoxic effect of unconjugated bilirubin in human neuroblastoma SH-SY5Y cells is modulated by the expression level of MRP1 but not MDR1. Biochem. J..

[19] Kaur B., Sohrabi Y., Achreja A., Lisanti M.P., Martinez-Outschoorn U.E. (2023). Hallmark of cancer: Reprogramming of cellular metabolism. Front. Media SA.

[20] Deaver J.W., López S.M., Ryan P.J., Nghiem P.P., Riechman S.E., Fluckey J.D. (2020). Regulation of cellular anabolism by mTOR: Or how I learned to stop worrying and love translation. Sports Med. Health Sci..

[21] Zhang N., Gao M., Wang Z., Zhang J., Cui W., Li J., Zhu X., Zhang H., Yang D.-H., Xu X. (2021). Curcumin reverses doxorubicin resistance in colon cancer cells at the metabolic level. J. Pharm. Biomed. Anal..

[22] Pawar C.S., Balamurugan K., Baskar S., Prasad N.R., Khan H.A. (2025). Enhancing chemosensitivity in drug-resistant breast cancer cells using β-cyclodextrin-loaded quercetin and doxorubicin inclusion complex via modulating SRC/PI3K/akt pathway. Appl. Biochem. Biotechnol..

[23] Mirzaei S., Gholami M.H., Zabolian A., Saleki H., Bagherian M., Torabi S.M., Sharifzadeh S.O., Hushmandi K., Fives K.R., Khan H. (2023). Resveratrol augments doxorubicin and cisplatin chemotherapy: A novel therapeutic strategy. Curr. Mol. Pharmacol..

[24] Liu Y., Fang M., Tu X., Mo X., Zhang L., Yang B., Wang F., Kim Y.-B., Huang C., Chen L. (2024). Dietary Polyphenols as Anti-Aging Agents: Targeting the Hallmarks of Aging. Nutrients.

[25] Shakoor H., Feehan J., Apostolopoulos V., Platat C., Al Dhaheri A.S., Ali H.I., Ismail L.C., Bosevski M., Stojanovska L. (2021). Immunomodulatory effects of dietary polyphenols. Nutrients.

[26] Cao H., Ou J., Chen L., Zhang Y., Szkudelski T., Delmas D., Daglia M., Xiao J. (2019). Dietary polyphenols and type 2 diabetes: Human Study and Clinical Trial. Crit. Rev. Food Sci. Nutr..

[27] Patra S., Pradhan B., Nayak R., Behera C., Das S., Patra S.K., Efferth T., Jena M., Bhutia S.K. (2021). Dietary polyphenols in chemoprevention and synergistic effect in cancer: Clinical evidences and molecular mechanisms of action. Phytomedicine.

[28] Ahmad N., Qamar M., Yuan Y., Nazir Y., Wilairatana P., Mubarak M.S. (2022). Dietary polyphenols: Extraction, identification, bioavailability, and role for prevention and treatment of colorectal and prostate cancers. Molecules.

[29] Shuvalov O., Daks A., Fedorova O., Petukhov A., Barlev N. (2021). Linking metabolic reprogramming, plasticity and tumor progression. Cancers.

[30] Nong S., Han X., Xiang Y., Qian Y., Wei Y., Zhang T., Tian K., Shen K., Yang J., Ma X. (2023). Metabolic reprogramming in cancer: Mechanisms and therapeutics. MedComm.

[31] Shuvalov O., Petukhov A., Daks A., Fedorova O., Vasileva E., Barlev N.A. (2017). One-carbon metabolism and nucleotide biosynthesis as attractive targets for anticancer therapy. Oncotarget.

[32] Tjaden B., Baum K., Marquardt V., Simon M., Trajkovic-Arsic M., Kouril T., Siebers B., Lisec J., Siveke J.T., Schulte J.H. (2020). N-Myc-induced metabolic rewiring creates novel therapeutic vulnerabilities in neuroblastoma. Sci. Rep..

[33] Pouliou M., Koutsi M.A., Champezou L., Giannopoulou A.-I., Vatsellas G., Piperi C., Agelopoulos M. (2023). MYCN amplifications and metabolic rewiring in neuroblastoma. Cancers.

[34] Jahangiri L. (2024). Metabolic targeting of neuroblastoma, an update. Cancer Lett..

[35] Nazarov A., Parfenyev S., Shuvalov O., Frolova K., Naminat E., Nevzorov I., Petukhov A., Karpova N., Fedorova O., Barlev N. (2025). Effects of n-Myc and c-Myc on the expression of p53 family members and their transcriptional targets in human neuroblastoma cells. Biochem. Biophys. Res. Commun..

[36] Cormerais Y., Lapp S.C., Kalafut K.C., Cissé M.Y., Shin J., Stefadu B., Personnaz J., Schrötter S., Freed J., D’Amore A. (2025). AKT-mediated phosphorylation of TSC2 controls stimulus-and tissue-specific mTORC1 signaling and organ growth. Dev. Cell.

[37] Glaviano A., Foo A.S., Lam H.Y., Yap K.C., Jacot W., Jones R.H., Eng H., Nair M.G., Makvandi P., Geoerger B. (2023). PI3K/AKT/mTOR signaling transduction pathway and targeted therapies in cancer. Mol. Cancer.

[38] Kierans S.J., Taylor C.T. (2024). Glycolysis: A multifaceted metabolic pathway and signalling hub. J. Biol. Chem..

[39] Shuvalov O., Kirdeeva Y., Fefilova E., Daks A., Fedorova O., Parfenyev S., Nazarov A., Vlasova Y., Krasnov G.S., Barlev N.A. (2024). 20-Hydroxyecdysone Boosts Energy Production and Biosynthetic Processes in Non-Transformed Mouse Cells. Antioxidants.

[40] Majumder A., Bano S., Nayak K.B. (2024). The Pivotal Role of One-Carbon Metabolism in Neoplastic Progression During the Aging Process. Biomolecules.

[41] Zhao E., Hou J., Cui H. (2020). Serine–glycine-one-carbon metabolism: Vulnerabilities in MYCN-amplified neuroblastoma. Oncogenesis.

[42] Wang T., Liu L., Chen X., Shen Y., Lian G., Shah N., Davidoff A.M., Yang J., Wang R. (2018). MYCN drives glutaminolysis in neuroblastoma and confers sensitivity to an ROS augmenting agent. Cell Death Dis..

[43] Ruiz-Pérez M.V., Sainero-Alcolado L., Oliynyk G., Matuschek I., Balboni N., Ubhayasekera S.K.A., Snaebjornsson M.T., Makowski K., Aaltonen K., Bexell D. (2021). Inhibition of fatty acid synthesis induces differentiation and reduces tumor burden in childhood neuroblastoma. IScience.

[44] Kling M.J., Griggs C.N., McIntyre E.M., Alexander G., Ray S., Challagundla K.B., Joshi S.S., Coulter D.W., Chaturvedi N.K. (2021). Synergistic efficacy of inhibiting MYCN and mTOR signaling against neuroblastoma. BMC Cancer.

[45] Gupta S.C., Prasad S., Kim J.H., Patchva S., Webb L.J., Priyadarsini I.K., Aggarwal B.B. (2011). Multitargeting by curcumin as revealed by molecular interaction studies. Nat. Prod. Rep..

[46] Liczbiński P., Michałowicz J., Bukowska B. (2020). Molecular mechanism of curcumin action in signaling pathways: Review of the latest research. Phytother. Res..

[47] Mahmoudi A., Jamialahmadi T., Kesharwani P., Sahebkar A. (2024). Bioinformatic analysis of the molecular targets of curcumin in colorectal cancer. Pathol. Res. Pract..

[48] Ren Z.Q., Zheng S.Y., Sun Z., Luo Y., Wang Y.T., Yi P., Li Y.S., Huang C., Xiao W.F. (2025). Resveratrol: Molecular Mechanisms, Health Benefits, and Potential Adverse Effects. MedComm.

[49] Boo H.J., Yoon D., Choi Y., Kim Y., Cha J.S., Yoo J. (2025). Quercetin: Molecular Insights into Its Biological Roles. Biomolecules.

[50] Siddiqui F.A., Prakasam G., Chattopadhyay S., Rehman A.U., Padder R.A., Ansari M.A., Irshad R., Mangalhara K., Bamezai R.N., Husain M. (2018). Curcumin decreases Warburg effect in cancer cells by down-regulating pyruvate kinase M2 via mTOR-HIF1α inhibition. Sci. Rep..

[51] Bianchi G., Ravera S., Traverso C., Amaro A., Piaggio F., Emionite L., Bachetti T., Pfeffer U., Raffaghello L. (2018). Curcumin induces a fatal energetic impairment in tumor cells in vitro and in vivo by inhibiting ATP-synthase activity. Carcinogenesis.

[52] Nocito M.C., Avena P., Zavaglia L., De Luca A., Chimento A., Hamad T., La Padula D., Stancati D., Hantel C., Sirianni R. (2023). Adrenocortical Carcinoma (ACC) Cells Rewire Their Metabolism to Overcome Curcumin Antitumoral Effects Opening a Window of Opportunity to Improve Treatment. Cancers.

[53] Yang R., Fang X.-L., Zhen Q., Chen Q.-Y., Feng C. (2019). Mitochondrial targeting nano-curcumin for attenuation on PKM2 and FASN. Colloids Surf. B Biointerfaces.

[54] Li W., Ma X., Li N., Liu H., Dong Q., Zhang J., Yang C., Liu Y., Liang Q., Zhang S. (2016). Resveratrol inhibits Hexokinases II mediated glycolysis in non-small cell lung cancer via targeting Akt signaling pathway. Exp. Cell Res..

[55] Wu H., He L., Shi J., Hou X., Zhang H., Zhang X., An Q., Fan F. (2018). Resveratrol inhibits VEGF-induced angiogenesis in human endothelial cells associated with suppression of aerobic glycolysis via modulation of PKM 2 nuclear translocation. Clin. Exp. Pharmacol. Physiol..

[56] Rodríguez-Enríquez S., Pacheco-Velázquez S.C., Marín-Hernández Á., Gallardo-Pérez J.C., Robledo-Cadena D.X., Hernández-Reséndiz I., García-García J.D., Belmont-Díaz J., López-Marure R., Hernández-Esquivel L. (2019). Resveratrol inhibits cancer cell proliferation by impairing oxidative phosphorylation and inducing oxidative stress. Toxicol. Appl. Pharmacol..

[57] Zhang P., Li H., Wu M.-L., Chen X.-Y., Kong Q.-Y., Wang X.-W., Sun Y., Wen S., Liu J. (2006). c-Myc downregulation: A critical molecular event in resveratrol-induced cell cycle arrest and apoptosis of human medulloblastoma cells. J. Neuro-Oncol..

[58] Asgharian P., Tazekand A.P., Hosseini K., Forouhandeh H., Ghasemnejad T., Ranjbar M., Hasan M., Kumar M., Beirami S.M., Tarhriz V. (2022). Potential mechanisms of quercetin in cancer prevention: Focus on cellular and molecular targets. Cancer Cell Int..

[59] Chan C.-Y., Hong S.-C., Chang C.-M., Chen Y.-H., Liao P.-C., Huang C.-Y. (2023). Oral squamous cell carcinoma cells with acquired resistance to erlotinib are sensitive to anti-cancer effect of quercetin via pyruvate kinase M2 (PKM2). Cells.

[60] Jia L., Huang S., Yin X., Zan Y., Guo Y., Han L. (2018). Quercetin suppresses the mobility of breast cancer by suppressing glycolysis through Akt-mTOR pathway mediated autophagy induction. Life Sci..

[61] Sturza A., Pavel I., Ancușa S., Danciu C., Dehelean C., Duicu O., Muntean D. (2018). Quercetin exerts an inhibitory effect on cellular bioenergetics of the B164A5 murine melanoma cell line. Mol. Cell. Biochem..

[62] Zhao P., Mao J.M., Zhang S.Y., Zhou Z.Q., Tan Y., Zhang Y. (2014). Quercetin induces HepG2 cell apoptosis by inhibiting fatty acid biosynthesis. Oncol. Lett..

[63] Zenkov R.G., Kirsanov K.I., Ogloblina A.M., Vlasova O.A., Naberezhnov D.S., Karpechenko N.Y., Fetisov T.I., Lesovaya E.A., Belitsky G.A., Dolinnaya N.G. (2022). Effects of G-quadruplex-binding plant secondary metabolites on c-MYC expression. Int. J. Mol. Sci..

[64] Ye H., Zhang H., Xiang J., Shen G., Yang F., Wang F., Wang J., Tang Y. (2024). Advances and prospects of natural dietary polyphenols as G-quadruplex stabilizers in biomedical applications. Int. J. Biol. Macromol..

[65] Ribeiro E., Vale N. (2024). The Role of Resveratrol in Cancer Management: From Monotherapy to Combination Regimens. Targets.

[66] Brown K., Theofanous D., Britton R.G., Aburido G., Pepper C., Sri Undru S., Howells L. (2024). Resveratrol for the management of human health: How far have we come? A systematic review of resveratrol clinical trials to highlight gaps and opportunities. Int. J. Mol. Sci..

[67] Mirza M.A., Mahmood S., Hilles A.R., Ali A., Khan M.Z., Zaidi S.A.A., Iqbal Z., Ge Y. (2023). Quercetin as a therapeutic product: Evaluation of its pharmacological action and clinical applications—A review. Pharmaceuticals.

[68] Gupta S.C., Patchva S., Aggarwal B.B. (2013). Therapeutic roles of curcumin: Lessons learned from clinical trials. AAPS J..

[69] Elgar K. (2022). Curcumin: A review of clinical use and efficacy. Nutr. Med. J..

[70] Mansouri K., Rasoulpoor S., Daneshkhah A., Abolfathi S., Salari N., Mohammadi M., Rasoulpoor S., Shabani S. (2020). Clinical effects of curcumin in enhancing cancer therapy: A systematic review. BMC Cancer.

[71] Tu K.X., Ou Q.J., Lin F.T., Zhao Y.T., Zhou R.H., Zhou R.L., Fang Y.J., Zhang C.X. (2025). Higher Intake of Resveratrol Is Associated with a Lower Risk of Colorectal Cancer: A Large-Scale Case–Control Study. Phytother. Res..

[72] Cai H., Scott E., Kholghi A., Andreadi C., Rufini A., Karmokar A., Britton R.G., Horner-Glister E., Greaves P., Jawad D. (2015). Cancer chemoprevention: Evidence of a nonlinear dose response for the protective effects of resveratrol in humans and mice. Sci. Transl. Med..

[73] Perkins S., Verschoyle R.D., Hill K., Parveen I., Threadgill M.D., Sharma R.A., Williams M.L., Steward W.P., Gescher A.J. (2002). Chemopreventive efficacy and pharmacokinetics of curcumin in the min/+ mouse, a model of familial adenomatous polyposis. Cancer Epidemiol. Biomark. Prev..

[74] Liu Y., Wu Y.-M., Zhang P.-Y. (2015). Protective effects of curcumin and quercetin during benzo (a) pyrene induced lung carcinogenesis in mice. Eur. Rev. Med. Pharmacol. Sci..

[75] Del Bo’ C., Bernardi S., Marino M., Porrini M., Tucci M., Guglielmetti S., Cherubini A., Carrieri B., Kirkup B., Kroon P. (2019). Systematic review on polyphenol intake and health outcomes: Is there sufficient evidence to define a health-promoting polyphenol-rich dietary pattern?. Nutrients.

[76] Mérida D.M., Vitelli-Storelli F., Moreno-Franco B., Rodríguez-Ayala M., López-García E., Banegas J.R., Rodríguez-Artalejo F., Guallar-Castillón P. (2023). Polyphenol intake and mortality: A nationwide cohort study in the adult population of Spain. Clin. Nutr..

[77] Zupo R., Castellana F., Lisco G., Corbo F., Crupi P., Sardone R., Panza F., Lozupone M., Rondanelli M., Clodoveo M.L. (2024). Dietary Intake of Polyphenols and All-Cause Mortality: A Systematic Review with Meta-Analysis. Metabolites.

[78] Kishino M., Kanehara R., Mori N., Ishihara J., Takachi R., Yamaji T., Iwasaki M., Tsugane S., Sawada N. (2025). Dietary polyphenol intake and risk of overall and site-specific cancers: The Japan Public Health Center-based Prospective Study. J. Nutr..

[79] Fan L., Fike L.T., Munro H., Yu D., Si H., Shrubsole M.J., Dai Q. (2025). Dietary polyphenols and risk of breast cancer in a predominantly low-income population: A prospective analysis in the Southern Community Cohort Study (SCCS). Am. J. Clin. Nutr..

[80] Shuvalov O., Kirdeeva Y., Fefilova E., Netsvetay S., Zorin M., Vlasova Y., Fedorova O., Daks A., Parfenyev S., Barlev N. (2023). 20-Hydroxyecdysone Confers Antioxidant and Antineoplastic Properties in Human Non-Small Cell Lung Cancer Cells. Metabolites.

