# Supplementary figures and images for: Dietary Polyphenol Combinations Have a Multifaceted Inhibitory Effect on Metabolic Rewiring and Signaling Pathways in Neuroblastoma
Source: Pharmaceuticals (Basel). 2025 Nov 12;18(11):1717. doi: 10.3390/ph18111717 (PMC12655026; doi:10.3390/ph18111717)

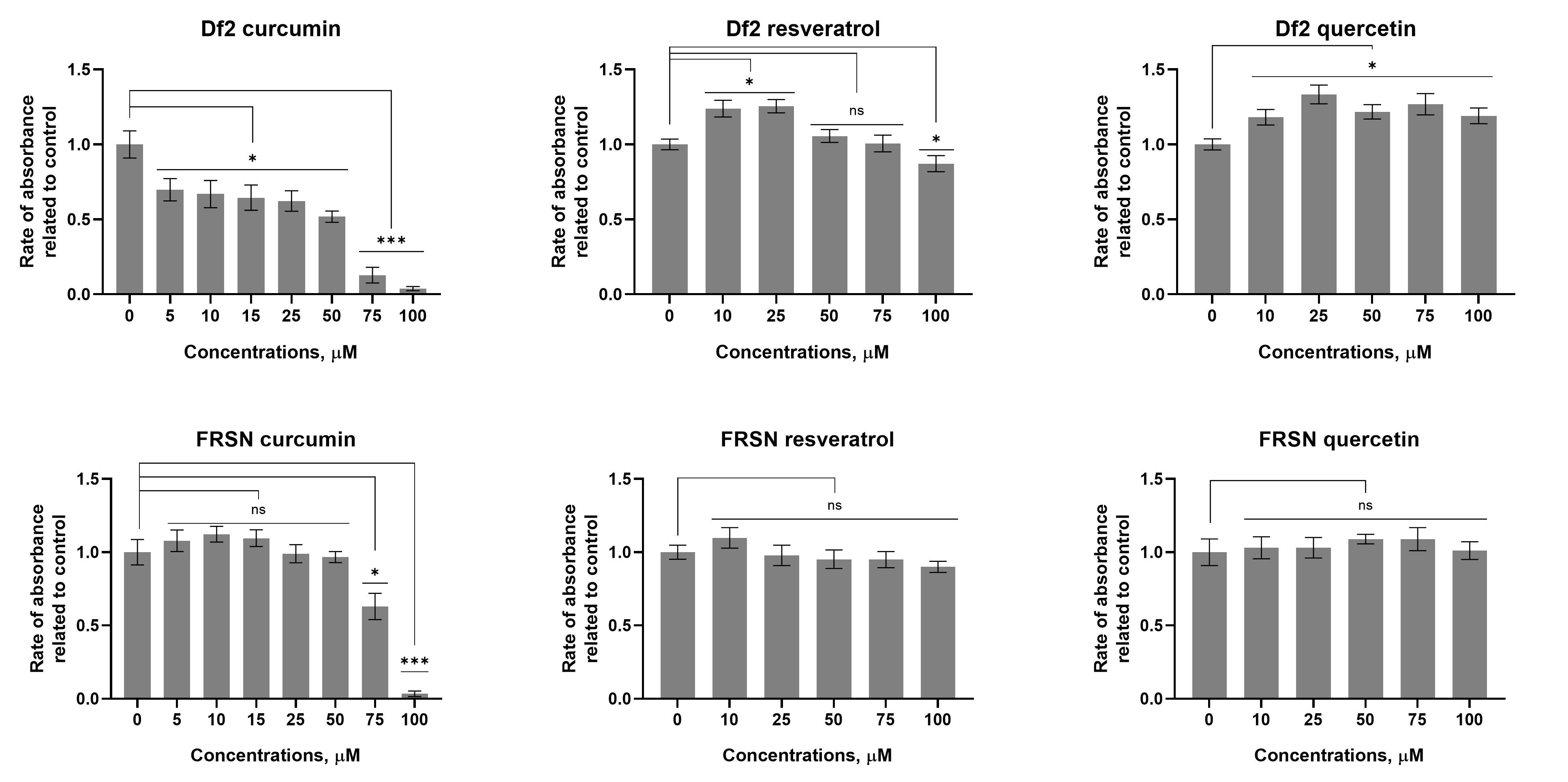

Supplement: Supplementary file 1 [file pharmaceuticals-18-01717-s001.zip › Figure S1.jpg]

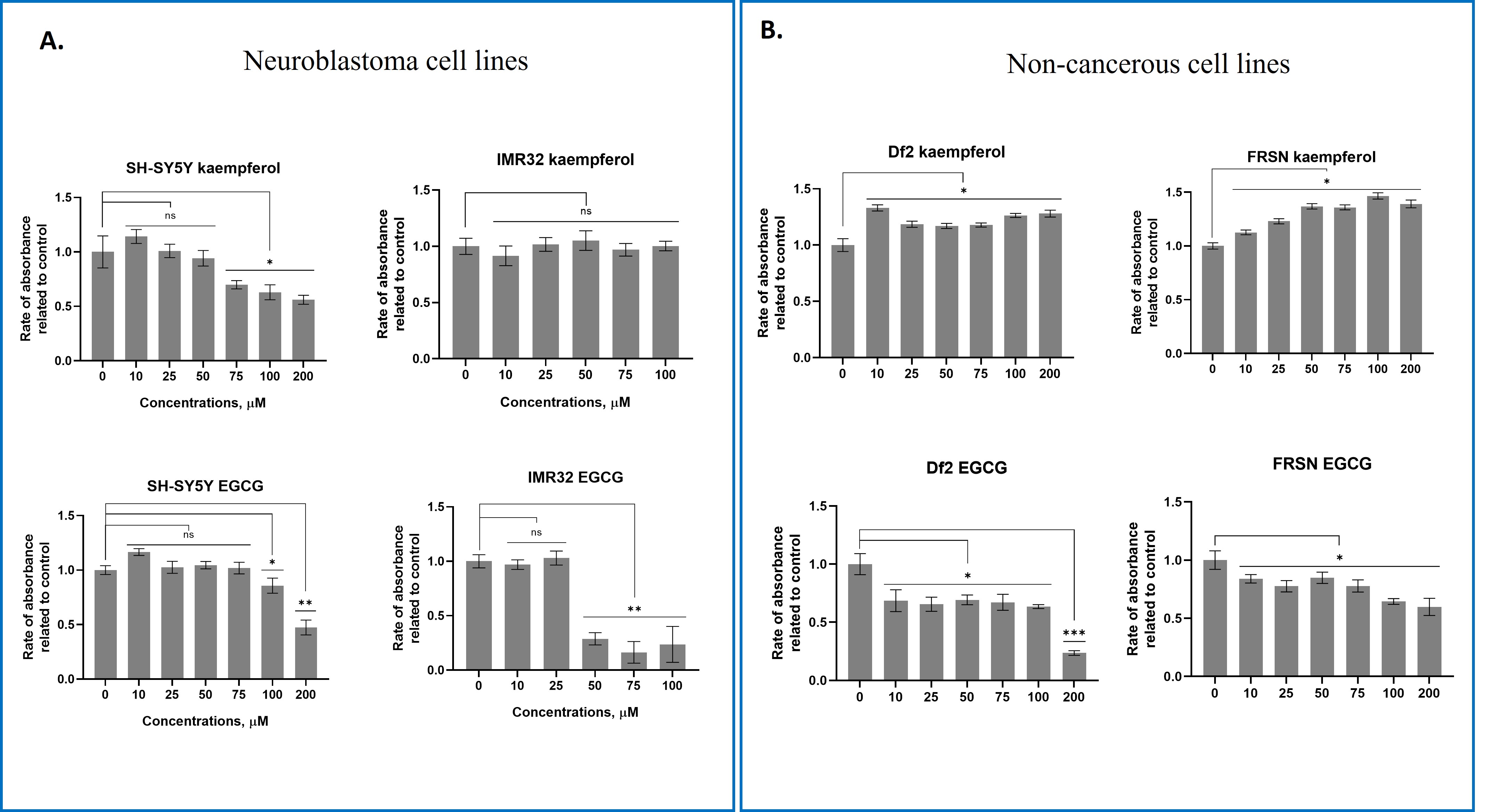

Supplement: Supplementary file 1 [file pharmaceuticals-18-01717-s001.zip › Figure S2.jpg]

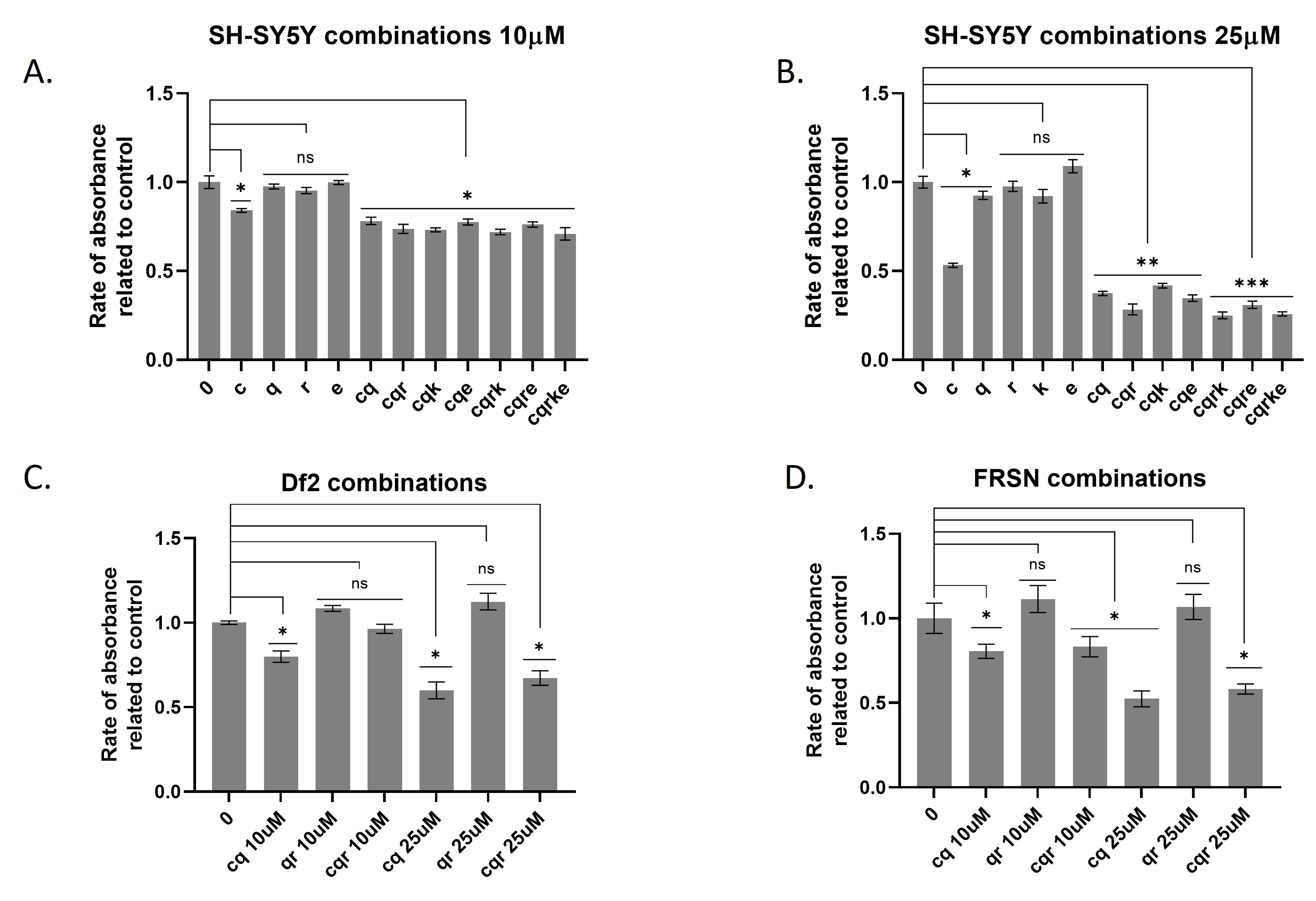

Supplement: Supplementary file 1 [file pharmaceuticals-18-01717-s001.zip › Figure S3.jpg]

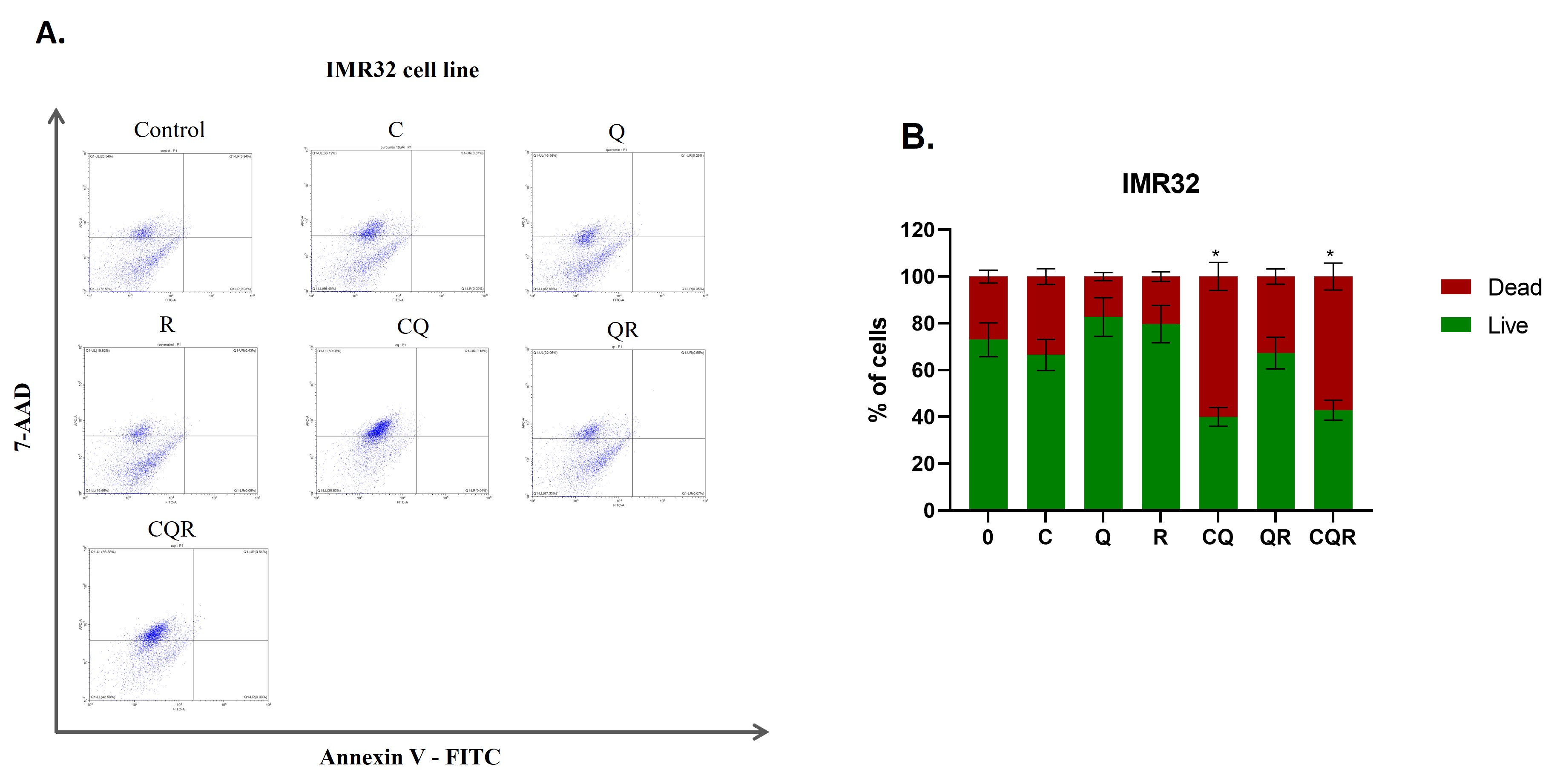

Supplement: Supplementary file 1 [file pharmaceuticals-18-01717-s001.zip › Figure S4.jpg]

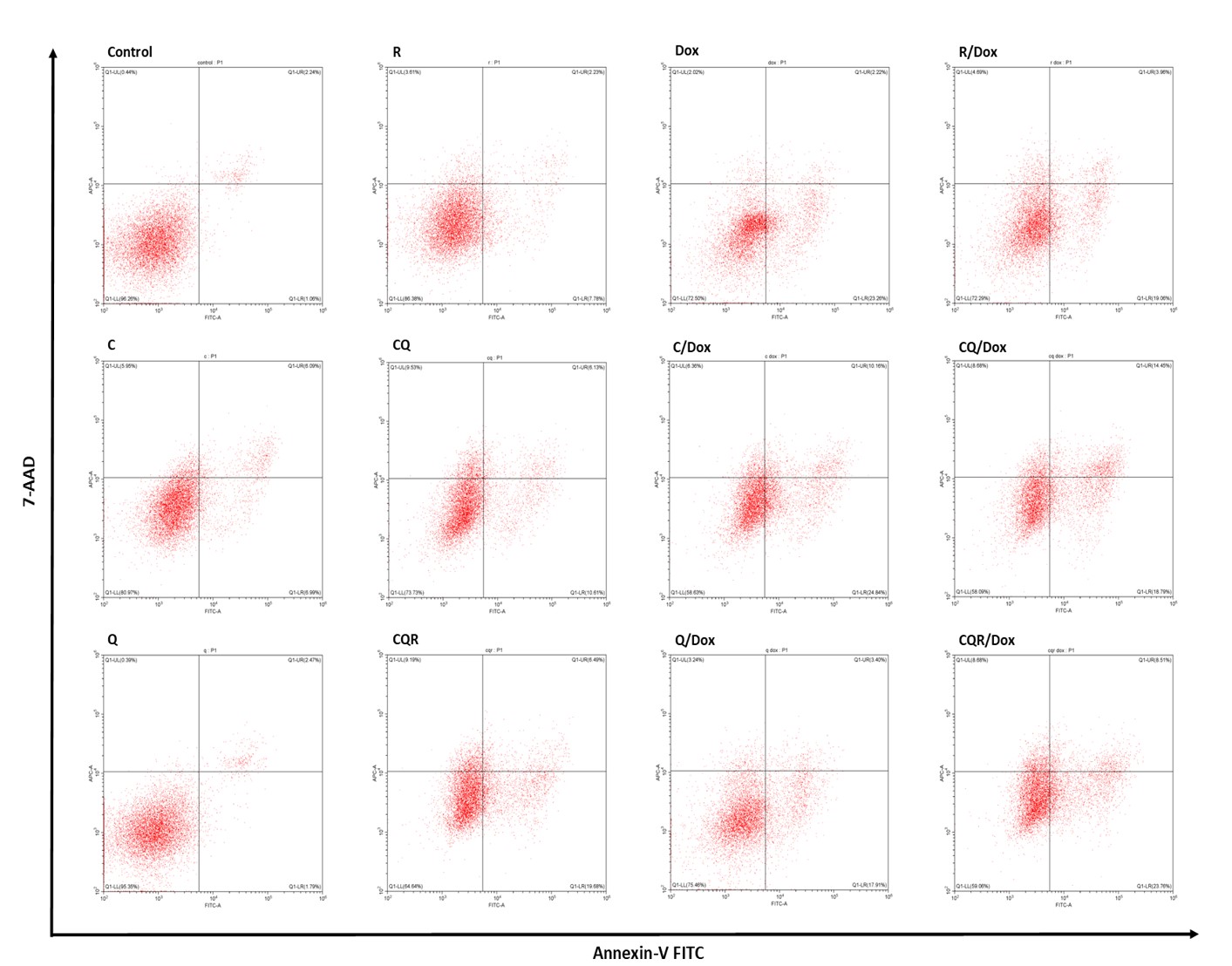

Supplement: Supplementary file 1 [file pharmaceuticals-18-01717-s001.zip › Figure S5.jpg]
